# Pre-Gestational Consumption of Ultra-Processed Foods and Risk of Gestational Diabetes in a Mediterranean Cohort. The SUN Project

**DOI:** 10.3390/nu13072202

**Published:** 2021-06-26

**Authors:** Alessandro Leone, Miguel Ángel Martínez-González, Winston Craig, Ujué Fresán, Clara Gómez-Donoso, Maira Bes-Rastrollo

**Affiliations:** 1 International Center for the Assessment of Nutritional Status, Department of Food, Environmental and Nutritional Sciences, University of Milan, 20133 Milan, Italy; 2 Department of Preventive Medicine and Public Health, School of Medicine, University of Navarra, 31009 Pamplona, Spain; mamartinez@unav.es (M.Á.M.-G.); cgomezd@unav.es (C.G.-D.); mbes@unav.es (M.B.-R.); 3 CIBERobn, Instituto de Salud Carlos III, 28029 Madrid, Spain; 4 Navarra Institute for Health Research (IdiSNA), 31009 Pamplona, Spain; 5 Department of Nutrition, TH Chan School of Public Health, Harvard University, Boston, MA 02115, USA; 6 School of Public Health, Loma Linda University, Loma Linda, CA 92354, USA; hikingut.co4me@gmail.com; 7 ISGlobal, 08036 Barcelona, Spain; ujuefresan@gmail.com

**Keywords:** gestational diabetes, ultra-processed food, pregnancy, cohort study

## Abstract

We aimed to investigate the relationship between the pre-gestational consumption of ultra-processed foods (UPF) and the risk of gestational diabetes (GDM). We carried out a prospective study among 3730 Spanish women of the SUN cohort who reported at least one pregnancy after baseline recruitment. Cases of GDM were identified among women with a confirmed diagnosis of GDM. UPF consumption was assessed through a validated, semi-quantitative food frequency questionnaire and the frequency of UPF consumption was categorized in tertiles. We identified 186 cases of GDM. In the pooled sample, we did not observe a significant association of UPF with the risk of GDM. When we stratified by age, the multivariate OR for the third tertile of UPF consumption compared with the lowest one was 2.05 (95% CI 1.03, 4.07) in women aged ≥30 years at baseline (*P*_trend_ = 0.041). The association remained significant in a sensitivity analysis after changing many of our assumptions and adjusting for additional confounders. No association between a higher UPF consumption and GDM risk was observed in women aged 18–29 years. The pre-gestational UPF consumption may be a risk factor for GDM, especially in women aged 30 years or more. Confirmatory studies are needed to validate these findings.

## 1. Introduction

Gestational diabetes mellitus (GDM) is a condition characterized by glucose intolerance resulting in hyperglycemia that develops during pregnancy [1]. GDM has been found to be associated with many adverse pregnancy outcomes, such as macrosomia, preeclampsia, large for gestational age infants, perinatal mortality, and Caesarean delivery [2]. Moreover, GDM is a risk factor for future adverse health outcomes for mothers and their children. Women who develop GDM have an increased risk of Type 2 diabetes, cardiovascular morbidity, metabolic syndrome, and cancer, while children have an increased risk of obesity, impaired glucose tolerance, and vascular disorders [3].

In Europe, the prevalence of GDM is, on average, 5.4% [4] but it is expected to continue to rise as a result of the increasing prevalence of overweight and obesity in women [5,6], unhealthy diet, and sedentary lifestyles [7].

Dietary habits before pregnancy have been found to be associated with the risk of GDM. Consistent evidence suggests that certain dietary patterns, especially those richer in fruits, vegetables, wholegrains, legumes, nuts, and fish and poorer in red and processed meats are associated with a reduced risk of GDM, whereas Western-style dietary patterns, characterized by a higher consumption of refined grains, red and processed meat, and whole-fat dairy products and lower intakes of fruits and vegetables, would seem to be associated with an increased risk of GDM [8,9].

As part of a Western dietary pattern, the consumption of ultra-processed foods (UPF) could contribute to the risk of GDM. These are convenient, hyperpalatable, and highly profitable food formulations in which whole foods are mostly or entirely absent [10]. The consumption of these food products has increased dramatically in many countries over the past two decades, accounting now for 54%, 58%, and 48% of daily calories consumed in the UK, USA, and Canada, respectively [11,12,13].

From a nutritional point of view, UPF are high in fat, sugar, and salt and low in fiber, and, for this reason, may be an important driver of the increasing prevalence of obesity and diabetes. High UPF consumption has been found to be linked to the risk of obesity and diabetes in several prospective cohorts [14,15,16,17]. Recent clinical trials have also shown that UPF consumption causes excessive energy intake and weight gain [18] and a greater accumulation of visceral fat [19], a known risk factor for insulin resistance [20]. Furthermore, UPF have a higher impact on glycemic response compared with unprocessed foods [21] and contain some chemical compounds that have been shown to be associated with increased glucose intolerance and insulin resistance [22].

Pregnancy itself is a physiological state of the woman characterized by insulin resistance [23]. Thus, it is possible that pregnancy exacerbates a pre-existing condition of insulin resistance, leading to the development of GDM. A recent study supported this hypothesis, showing that UPF consumption during the third trimester influenced glycemic control in pregnant women with pre-existing diabetes [24].

We are not aware of previous prospective studies investigating the specific role of UPF consumption on the incidence of GDM. Therefore, we conducted the present analysis in women of the SUN cohort to appraise whether UPF consumption during reproductive age was associated with the development of GDM during pregnancy.

## 2. Materials and Methods

### 2.1. Study Design and Participants

The SUN project is an ongoing, prospective, and dynamic cohort that began in 1999 and aimed to investigate the associations of dietary habits and lifestyle with many health outcomes. The cohort includes former graduates of the University of Navarra, Spanish-registered professionals, and other university graduates. Baseline information on dietary habits, lifestyles, and health conditions was obtained using mailed or emailed questionnaires. Information regarding health outcomes was updated every 2 years through follow-up questionnaires. Dietary habits were updated after 10 years of follow-up. Along with the baseline questionnaire, participants received a fact sheet about the study program, including information about the specific data that would be required by future questionnaires, the protections to safeguard their privacy, and the future feedback from the research team. All potential candidates were also informed about their right to refuse to participate in the SUN study or to withdraw their consent to participate at any time without reprisal. This study was conducted in accordance with the guidelines laid down in the Declaration of Helsinki. The Human Research Ethical Committee at the University of Navarra approved all the study procedures (091/2008).

**Figure 1:**
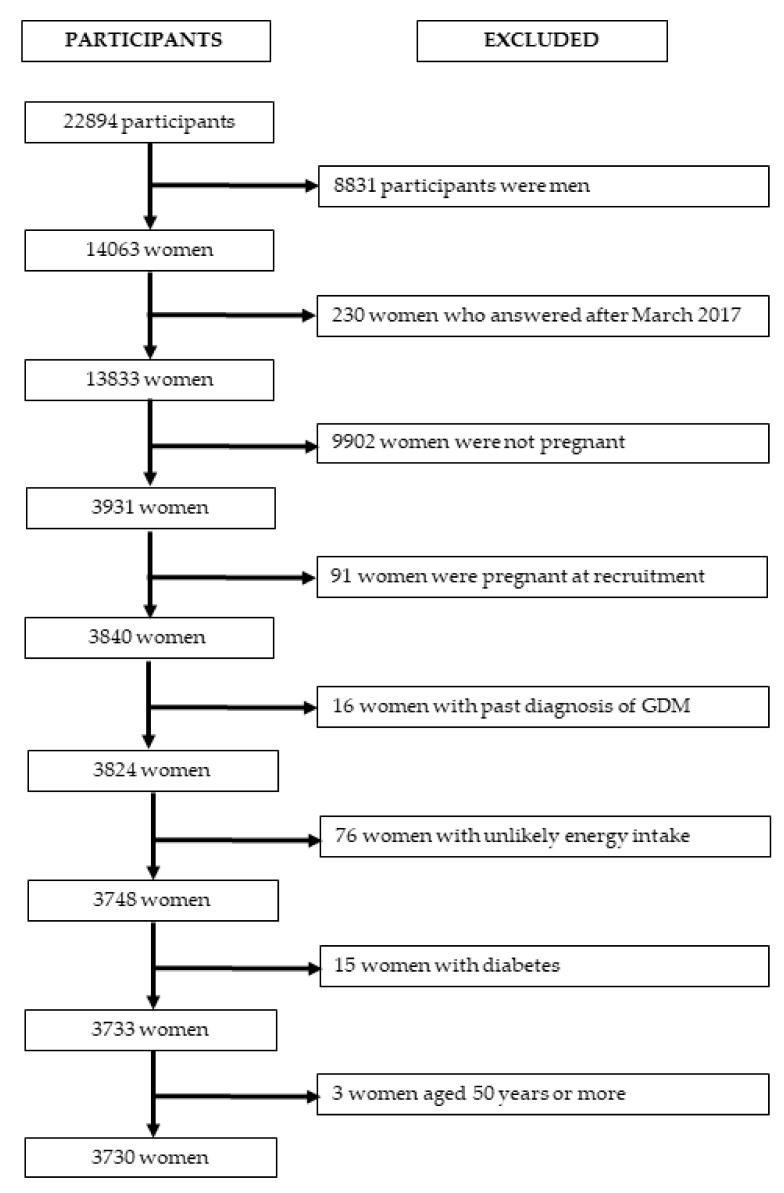
Flow chart showing the selection process of participants in the SUN project to be included in the present analysis. Energy intake below the first percentile or above the 99th percentile was considered unlikely. Abbreviation: GDM, gestational diabetes mellitus.

For this study, we used the version of the database updated in December 2019. Out of a total of 22,894 participants, we excluded 8831 men; 230 women who responded the baseline questionnaire after March 2017, as they have not been in the cohort for enough time to be able to respond to at least the first follow-up questionnaire; 9902 women not reporting any pregnancy during follow-up; 91 women who were pregnant at baseline; 16 prevalent cases of GDM; 76 women reporting unlikely energy intake (<1st percentile or >99th percentile); 15 prevalent cases of Type 1 or 2 diabetes; and 3 women aged 50 years or more (Figure 1). The final database included 3730 women reporting at least 1 pregnancy during follow-up.

### 2.2. Exposure Assessment

A semi-quantitative food frequency questionnaire (FFQ, 136 food items) previously validated and repeatedly reevaluated in Spain [25,26,27] was used to assess dietary habits at baseline and again after 10 years of follow-up. For each food item, the FFQ included a typical portion size. We measured the frequencies of consumption in 9 categories, ranging from never or almost never to more than 6 servings daily. We multiplied the portion size by the frequency of consumption in order to estimate daily consumption for each food item. All foods and beverages were then classified according to the NOVA classification (Table 1) [28].

UPF are industrial formulations made predominantly or entirely from substances extracted from foods, derived from food constituents, or chemically synthesized from food substrates or other organic sources. Examples include processed meat, sausages, biscuits and pastries, chocolate and candies, sweet or savory packaged snacks, instant packaged soups and noodles, fruit yogurts, carbonated drinks, and sugared milk and fruit drinks. The frequency of UPF consumption was estimated by summing that of each food item included in the NOVA definition of UPF (a total of 34 items). To minimize the effect of variation in dietary habits over time, we used the mean consumption of UPF between baseline and at 10 years of follow-up. UPF consumption was then adjusted for energy intake through the residual method [29]. The sample was divided into tertiles according to the UPF frequency of consumption.

### 2.3. Outcome Assessment

Women were defined as a probable case of GDM if they reported a medical diagnosis of GDM in any of the follow-up questionnaires. If so, an additional questionnaire was sent requesting their medical reports. This additional questionnaire also inquired about previous glycemic disorders, the results of the OGTT test, and the indicated treatment. With this information, an endocrinologist, blinded to dietary exposure, adjudicated each case of new-onset GDM.

The diagnosis of GDM was made by following a 2-step procedure during Weeks 24–28 of gestation; the first step consisted of a 50 g oral glucose test with a cut-off of 140 mg/dL (7·8 mmol/L). Those who tested positive underwent a diagnostic 3 h 100 g oral glucose tolerance test with the cut-offs established in the Third Workshop—Conference on Gestational Diabetes Mellitus [30,31]: fasting plasma glucose, 105 mg/dL (5·8 mmol/L); 1-h value, 190 mg/dL (10.6 mmol/L); 2-h value, 165 mg/dL (9.2 mmol/L); 3-h value, 145 mg/dL (8.1 mmol/L). These criteria were applied to the population of the SUN project.

### 2.4. Covariates Assessment

We also obtained information about sociodemographic characteristics (e.g., sex and age), anthropometric variables (e.g., weight and height), lifestyle (e.g., smoking status, physical activity, time spent watching TV, and ongoing nutritional therapies), history of chronic diseases (e.g., cardiovascular disease, diabetes, and cancer), family history of diabetes, and parity using the baseline questionnaire. Self-reported weight and BMI were validated against objective measured height and weight in a sub-sample of this cohort, reporting high validity [32]. The validated Spanish version of the Harvard Nurses’ Health Study physical activity questionnaire, based on 17 items, was used to assess physical activity [33]. Leisure time activities were measured in metabolic equivalent tasks (METs) per week, assigning the usual energy expenditure to each activity, and multiplying by the time spent (in hours per week) for each activity. Energy and nutrient intake were also calculated on the basis of the information collected from the FFQ that was administered at baseline. Finally, a 9-point score of adherence to the Mediterranean diet was calculated according to Trichopoulou et al. [34].

### 2.5. Statistical Analysis

Many continuous variables did not follow a Gaussian distribution. Therefore, all are reported as 25th, 50th, and 75th percentiles. Discrete variables are reported as counts and percentages. Participants were categorized into tertiles of energy-adjusted UPF consumption (servings/day). A logistic regression model was fitted to assess the relationship of UPF consumption with the risk of GDM. Odds ratios (OR) and their 95% confidence intervals (95% CI) were calculated, including the exposure variable categorized into tertiles, considering the lowest tertile as the reference category. To control for potential confounding factors, we used a pre-specified multivariate model, selecting variables based on biological plausibility and in accordance with previous studies conducted in the SUN cohort [35,36,37,38,39] on this topic, as recommended by Hernan et al. [40]. The results were adjusted for age (continuous, years), BMI (continuous, kg/m^2^), education (discrete: 0 = diploma, 1 = college, 2 = postgraduate), smoking (discrete: 0 = not smoking, 1 = ex-smoker, 2 = smoker), physical activity (discrete, tertiles), family history of diabetes (discrete: 0 = no, 1 = yes, 2 = missing), recruitment year (discrete, quintiles), time between recruitment and first pregnancy or GDM (continuous), number of pregnancies during follow-up (discrete: 0 = 1, 1 = 2, 2 = ≥3), parity (discrete: 0 = nulliparous, 1 = 1–2 pregnancies, 2 = ≥3 pregnancies, 3 = missing), multiple pregnancy (discrete: 0 = no, 1 = yes), time spent watching TV (discrete, tertiles), hypertension (discrete: 0 = no, 1 = yes), following a nutritional therapy (discrete: 0 = no, 1 = yes, 2 = missing), and total energy intake (discrete, tertiles). Tests of linear trend across increasing categories of consumption were performed by assigning the median value to each category and treating it as a continuous variable. The linearity of continuous variables was tested using multivariable fractional polynomials. We also re-ran the analysis after stratifying for age at recruitment (18–29 years and ≥30 years) using age-specific tertiles of UPF consumption and confounders. Finally, we conducted a sensitivity analysis by changing some of our assumptions and adjusting for further confounders. A *p* value of <0.05 was considered statistically significant. Statistical analysis was performed using STATA version 12.0 (StataCorp).

## 3. Results

The baseline characteristics of the women in the SUN project, across tertiles of UPF consumption, are presented in Table 2.

Women had a median age 27 years (range: 18–49 years) at recruitment. The median consumption of UPF in our cohort was 3.9 servings/day, contributing to the 29.7% of total daily energy intake. Women who reported a higher UPF consumption were younger, less physically active, and with a higher BMI. Moreover, they were also more likely to be college graduates, active smokers, and nulliparous.

During a median follow-up of 7.2 years (range: 1–19 years), a total of 186 new confirmed cases of GDM were identified among 3730 pregnant women recruited in the SUN cohort, corresponding to 5.0% of the participants. GDM incidence among the tertiles of UPF consumption was 4.3, 5.7, and 5.0%.

In the multivariate model, we found that age (OR: 1.12; 95% CI: 1.08, 1.16 per 1-year increment), BMI (OR: 1.08; 95% CI: 1.02, 1.13 per 1 kg/m^2^ BMI increment), family history of diabetes (OR: 1.91; 95% CI: 1.27, 2.88), and having ≥3 pregnancies during follow-up (OR: 2.55; 95% CI: 1.56, 4.17) were risk factors for GDM. As regards the risk of GDM according to the tertiles of UPF consumption (Table 3), women in the third tertile had a 10% higher risk of GDM (OR: 1.10; 95% CI: 0.74, 1.64) than those in the lowest tertile. However, the association was not significant.

When we stratified our analysis by age, we observed that women aged ≥30 years with a higher UPF consumption presented a doubled risk for GDM compared with women with a lower consumption (OR: 2.05; 95% CI: 1.03, 4.07). Moreover, there was a significant dose–response relationship between the consumption of UPF and the risk of GDM (*P*_trend_ = 0.041). In contrast, UPF consumption was not associated with the risk of GDM in women aged 18–29 years.

Additionally, we conducted a sensitivity analysis by modifying some of our assumptions or introducing new potential confounders in order to assess the robustness of our results (Table 4). The results of the sensitivity analyses did not substantially change when the analysis was restricted to participants free of CVD and cancer, those not following a nutritional therapy, women at first pregnancy, or women who became pregnant within 10 years from recruitment. Changing the energy intake limits or further adjustment for adherence to the Mediterranean diet, or for carbohydrate and saturated fat intake did not change our results. When we used the proportion of UPF to total energy intake (%UPF) instead of the frequency of consumption, women aged 30 and older had a 52% increased risk of GDM, but the association was no longer significant. Similarly, when we used baseline UPF consumption for women reporting GDM or first pregnancy before 10 years of follow-up, statistical significance was lost.

## 4. Discussion

In this prospective cohort study including non-diabetic adult women, pre-gestational UPF consumption was associated with an increased risk of GDM in women aged 30 years or more but not in younger women. However, when we used the proportion of UPF to total energy intake as the exposure variable, women with higher UPF consumption kept showing a greater risk of GDM but the statistical significance was lost. This may depend on the different sensitivity of the energy adjustment method used. The residual energy-adjusted model may be more sensitive for detecting an association between dietary habits and the risk of disease than the nutrient density model, as the former is less influenced by the relationship between energy intake and disease [29]. Similarly, using the mean UPF consumption obtained by surveying dietary habits on two different occasions far apart in time may be more sensitive for detecting associations between food consumption and the incident disease risk than the baseline survey alone, as it may minimize dietary changes over time. This would explain why when we used only baseline dietary habits for women who had GDM and their first pregnancy during the first 10 years of follow-up, the association was lost.

Several previous observational prospective studies reported an increased risk of GDM associated with the pre-pregnancy consumption of certain food groups included in the UPF definition, such as processed red meat [39,41] and sugar-sweetened soft drinks [37]. Recently, a meta-analysis of observational studies found an overall increased GDM risk associated with higher adherence to the Western dietary pattern [9]. However, due to the limited amount of evidence and the high heterogeneity among the studies, it is still difficult to draw definitive conclusions [8,9]. UPF are a hallmark of Western dietary patterns, and their consumption has been associated with all-cause mortality [10] and many negative health outcomes, such as overweight and obesity [14], hypertension [42], cardiovascular events [43], diabetes [15,16,17], and cancer [44]. Recently, a cross-sectional study showed that women with a higher proportion of UPF in total energy intake had a greater risk of excess body weight, but not of GDM, compared with women with lower UPF consumption [45]. To our knowledge, ours is the first epidemiologic study using a prospective design to assess the association between UPF consumption and the risk of GDM.

Several hypotheses could explain our findings. Firstly, it is known that pregnancy is characterized by a state of insulin resistance, favored by a surge in local and placental hormones, which is overcome through compensatory insulin secretion by the pancreatic β-cells [23]. In addition, pregnancy is also characterized by an altered inflammatory profile compared with the non-pregnant state [46], and the excessive inflammatory response may turn into further impairment of insulin action and possibly insufficient β-cell compensation [47]. Pregnant women who develop GDM are thought to have decreased peripheral insulin sensitivity already present before pregnancy [48] and dysfunctional β-cells unable to balance the increased insulin requirements, resulting in hyperglycemia [49]. Generally, UPF are characterized by poorer nutritional quality than unprocessed or processed foods, as they tend to be richer in energy, fat, sugar, and sodium and poorer in fibers. It has been demonstrated that the consumption of UPF causes an increase in energy intake [18] and a higher glycemic response, partly due to their high content of free sugars and refined carbohydrates [21]. The chronic excess carbohydrate intake may induce expansion of adipose tissue and may favor ectopic fat deposition into the liver and skeletal muscle, leading to insulin resistance [50,51]. On the other hand, the high fat intake from UPF consumption also causes an increased level of circulating free fatty acids, inducing increased hepatic lipogenesis and gluconeogenesis, as well as decreased insulin clearance, resulting in hyperinsulinemia and further exacerbation of insulin resistance [52]. The consumption of UPF has also been shown to produce greater pro-inflammatory potential in the diet [53], suggesting that consumption of these products may generate a state of chronic low-grade inflammation, with a consequent increased risk of insulin resistance [54]. Similarly, it is known that iron in processed red meat has a strong pro-oxidant effect which promotes the creation of hydroxyl radicals, increasing oxidative stress [55]. The pancreatic β-cell is particularly sensitive to this type of stress, as it has a poor antioxidant capacity. This makes it more susceptible to oxidative damage, ultimately leading to impaired insulin synthesis [56]. Second, the carbohydrates and fats from ultra-processed foods could interact with genes known to decrease insulin secretion and increase insulin resistance, enhancing their expression [57]. Third, beyond the low overall nutritional quality, the packaging of UPF may include some materials in contact with the food, for which endocrine disruptor properties are known, such as phthalates and bisphenol A [58,59]. These compounds are thought to play a role in the development of obesity, insulin-resistance, and diabetes [58]; recently, some observational studies reported an association with the risk of GDM [60,61]. Fourth, the preservatives in processed meat and advanced glycation products from cooking animal-derived foods have been shown to contribute to insulin resistance [62,63]. Therefore, it is biologically plausible that the pre-gestational consumption of UPF led to a state of insulin resistance that resulted in GDM during pregnancy. It has also been shown that glucose-stimulated insulin secretion linearly decreases with age at a rate of 0.7% per year (7% per decade) in subjects with normal glucose tolerance, while it doubles in subjects with impaired glucose tolerance [64]. In addition, quantitative and qualitative changes in the immune system have been observed with aging. This phenomenon is accompanied by cytokine dysregulation, with increased pro-inflammatory cytokines and decreased anti-inflammatory cytokines, leading to a chronic low-grade inflammatory state, with a consequent increased risk of comorbidity, including insulin resistance [65]. This means that pregnant women aged ≥30 years are less able to compensate for the insulin resistance resulting from pregnancy than younger pregnant women, and this would explain the association between UPF consumption and the risk of GDM in pregnant women aged 30 years or more.

Our findings are relevant in the context of the increasing prevalence of diabetes, as it is known that women who develop GDM and children whose mothers had GDM during pregnancy are very likely to develop Type 2 diabetes in the future [3]. Our results reinforce the importance of promoting healthy and sustainable dietary habits, geared towards increasing adherence to high-quality dietary patterns, such as the Mediterranean diet, as they have been found to be associated with lower odds of developing GDM during pregnancy [9].

Several strengths characterize the present study, including the prospective design, the large sample size with a >90% retention rate [66], the long follow-up, and the use of a FFQ that has been repeatedly validated in Spain [25,26,27]. In addition, we were able to control for several confounders, including family history of diabetes, nutritional status, and potential lifestyle and demographic confounders. In addition, we conducted several sensitivity analyses, and the results were robust. Moreover, all GDM diagnosis were confirmed by an endocrinologist, blinded to the exposure, using medical clinical records. Finally, the SUN cohort includes highly educated participants, and this means high-quality self-reported data.

That said, we are well aware that our study is not free of limitations. As we are dealing with a cohort of Spanish graduates, we could not assess the impact of ethnicity, but we can assume that almost all participants were Caucasian. Second, although an FFQ is probably the best method for assessing dietary habits in large cohort studies, it may be susceptible to some degree of measurement error. However, the FFQ used in the present study has been repeatedly validated and very frequently used with consistent results in many other external and independent cohorts [25,26,27]. Third, dietary habits were not investigated during pregnancy. It is possible that women changed their dietary habits once they became aware of their pregnant state. However, evidence has shown that consumption of sugar-sweetened beverages and fried food, considered as UPF, did not change between trimesters and their consumption was high [67]. Similarly, UPF consumption was recently found to be high even during pregnancy [68]. Fourth, the number of women aged 30 years or more who developed GDM was quite low. This may have affected the accuracy of the risk estimates, as evidenced by the rather wide confidence intervals. However, this did not prevent us from finding an association between UPD consumption and GDM risk. Fifth, the SUN project mainly includes middle-aged participants with a high education level. In our protocol, we applied this restriction to minimize potential confounding bias by education, socioeconomic status, disease, and presumed access to health care. Thus, the generalizability of our results should be based on common biological mechanisms instead of statistical representativeness. Further studies are required to confirm our results in pregnant women from other populations. Sixth, due to the observational nature of the present study, we cannot determine causation, and this study was unlikely to yield detailed evaluations of the underlying mechanisms. Finally, the diagnosis of GDM was based on a single time point, which did not allow us to differentiate early from late GDM or other underlying causes of glucose intolerance other than diet, including gestational weight gain or genetic predisposition.

## 5. Conclusions

In conclusion, our results suggest that a higher consumption of UPF during the reproductive age is an independent risk factor for GDM, especially in women aged 30 years or more. Further studies are needed to confirm these findings. Nevertheless, the promotion of dietary patterns characterized by minimally processed foods could protect against GDM.

## Figures and Tables

**Table 1:** Classification of foods in the SUN food frequency questionnaire according to the degree of processing (NOVA).

**Unprocessed or minimally processed foods** Vegetables, fruit, grains (white rice, pasta), legumes, milk (whole, semi-skimmed, and non-fat), meats, poultry, fish and seafood, eggs, fermented milk as yogurt, natural juice, coffee, and water
**Processed culinary ingredients** Vegetable oils (olive, sunflower, corn), butter, lard, chili, salt, sugar, and honey
**Processed foods** Breads (white and whole), cured traditional ham, bacon, condensed milk, cream, milk, cheeses, canned and bottled fruit, wine, and beer.
**Ultra-processed foods** Ice cream, petit-suisse, flan, pudding, custard, processed meat (chorizo, salami, mortadella, sausage, hamburger, morcilla), ham, spicy sausage/meatballs, pâté, foie-gras, potato chips, pizza, pre-prepared pies, breakfast cereals, margarine, cookies and chocolate cookies, doughnuts, muffins, croissants or other non-handmade pastries, cakes, churros, chocolates and candies, marzipan, nougat, carbonated drinks, artificially sugared beverages, milkshakes, fruit drinks, instant soups and creams, croquettes, mayonnaise, and alcoholic drinks produced by fermentation followed by distillation such as whisky, gin, and rum

**Table 2:** Characteristics of the recruited subjects across tertiles of energy-adjusted ultra-processed food consumption and in the total sample.

	Tertiles of Energy-Adjusted Ultra-Processed Food Consumption (servings/day)									
	Tertile 1			Tertile 2			Tertile 3			
	<3.3			3.3–4.5			>4.5			
	(*n* = 1244)			(*n* = 1243)			(*n* = 1243)			
	P25	P50	P75	P25	P50	P75	P25	P50	P75	*p*-Value
Age (years)	25	28	32	25	27	31	24	27	31	<0.001
BMI (kg/m^2^)	19.5	20.6	22.3	19.6	20.8	22.4	19.9	21.3	23.1	<0.001
Physical activity (METs/day)	4.6	14.7	29.3	3.4	13.7	26.7	3.3	13.3	26.2	0.005
Energy (kcal/day)	2070	2494	3019	1871	2265	2728	2012	2427	3005	<0.001
Vegetables (servings/day)	1.8	2.5	3.5	1.4	2	2.9	1.3	2	2.9	<0.001
Fruit (servings/day)	1.5	2.5	4	1.1	1.8	2.9	0.9	1.7	2.8	<0.001
Nuts (servings/day)	0.5	0.9	1.5	0.5	0.5	1	0.5	0.5	1	<0.001
Red and processed meat (servings/day)	1.6	2.3	3.1	1.7	2.3	3.1	1.8	2.4	3.3	0.005
Fish (servings/week)	3.4	4.9	7.4	2.9	4.3	6.3	2.9	3.9	6	<0.001
Cereals (servings/day)	1.2	1.7	2.8	1	1.4	2.2	1	1.5	2.4	<0.001
Legumes (servings/week)	1.9	2.5	3.5	1.5	2.4	3	1.4	2	3	<0.001
Milk and dairy products (servings/day)	2.3	3.3	4.5	2	2.9	4.1	2.1	3.1	4.3	<0.001
Olive oil (g/day)	10.7	25.2	29.5	8.9	12.1	25.8	9.5	13.2	26	<0.001
Alcohol (g/day)	0.6	2.1	4.5	0.6	2.1	4.9	0.6	2.2	6.1	0.086
Ultra-processed foods (servings/day)	2.1	2.7	3	3.6	3.9	4.2	4.9	5.5	6.5	<0.001
Ultra-processed foods/energy (%)	17.4	22.1	26.6	25.9	30.7	35.2	29.9	36.3	42.6	<0.001
	N	%		N	%		N	%		
Year of entrance in the cohort										0.032
1999–2000	339	27.3		370	29.8		338	27.2		
2001–2002	162	13		163	13.1		181	14.6		
2003–2004	210	16.9		229	18.4		258	20.8		
2005–2007	267	21.5		268	21.6		252	20.3		
2008–2017	266	21.4		213	17.1		214	17.2		
Education										0.019
Diploma	554	44.5		493	39.7		487	39.2		
Bachelor	634	51		693	55.8		711	57.2		
Postgraduate	56	4.5		57	4.6		45	3.6		
Smoking status										<0.001
Never	755	60.7		724	58.2		640	51.5		
Current	273	21.9		294	23.7		367	29.5		
Former	216	17.4		225	18.1		236	19		
Hypertension										0.194
No	1229	98.8		1232	99.1		1222	98.3		
Yes	15	1.2		11	0.9		21	1.7		
Following a nutritional therapy										<0.001
No	1135	91.2		1162	93.5		1113	89.5		
Yes	78	6.3		58	4.7		112	9		
Missing	31	2.5		23	1.9		18	1.4		
Family history of diabetes										0.138
No	1126	90.5		1118	89.9		1096	88.2		
Yes	118	9.5		125	10.1		147	11.8		
Parity										0.007
Nulliparous	969	77.9		1015	81.7		1031	82.9		
1–2 pregnancies	204	16.4		157	12.6		146	11.8		
≥3 pregnancies	33	2.7		44	3.5		32	2.6		
Missing	38	3.1		27	2.2		34	2.7		
Pregnancies during follow-up										0.287
1 pregnancy	561	45.1		511	41.1		550	44.3		
2 pregnancies	423	34		449	36.1		416	33.5		
≥3 pregnancies	260	20.9		283	22.8		277	22.3		
Gestational diabetes										0.287
No	1191	95.7		1172	94.3		1181	95		
Yes	53	4.3		71	5.7		62	5		

**Table 3:** Association of pre-gestational ultra-processed food consumption and risk of gestational diabetes in the pooled sample and stratified by age.

	Tertiles of Energy-Adjusted Ultra-Processed Food Consumption				
		T1	T2	T3	P for Trend
**Pooled sample**					
Ultra-processed foods	No. cases/total	53/1244	71/1243	62/1243	
	Median (servings/day)	2.7	3.9	5.5	
	Model 1 OR [95% CI]	Reference	1.36 [0.95, 1.96]	1.18 [0.81, 1.72]	0.474
	Model 2 OR [95% CI]	Reference	1.35 [0.94, 1.95]	1.13 [0.77, 1.65]	0.651
	Model 3 OR [95% CI]	Reference	1.41 [0.96, 2.06]	1.10 [0.74, 1.64]	0.818
**Women <30 years**					
Ultra-processed foods	No. cases/total	39/846	49/846	36/846	
	Median (servings/day)	2.8	3.9	5.6	
	Model 1 OR [95% CI]	Reference	1.27 [0.83, 1.96]	0.92 [0.58, 1.46]	0.607
	Model 2 OR [95% CI]	Reference	1.28 [0.84, 1.96]	0.89 [0.56, 1.41]	0.494
	Model 3 OR [95% CI]	Reference	1.25 [0.79, 1.98]	0.89 [0.54, 1.46]	0.524
**Women ≥30 years**					
Ultra-processed foods	No. cases/total	14/398	20/397	28/397	
	Median (servings/day)	2.5	3.8	5.4	
	Model 1 OR [95% CI]	Reference	1.46 [0.72, 2.92]	2.08 [1.08, 4.02]	0.025
	Model 2 OR [95% CI]	Reference	1.42 [0.70, 2.87]	1.94 [0.98, 3.81]	0.050
	Model 3 OR [95% CI]	Reference	1.56 [0.77, 3.15]	2.05 [1.03, 4.07]	0.041

Values are odd ratios (OR) and 95% confidence intervals. Model 1: unadjusted model; Model 2: model adjusted for age (continuous) and BMI (continuous); Model 3: model adjusted for age (continuous), BMI (continuous), education (discrete), smoking status (discrete), physical activity (tertiles), family history of diabetes (discrete), recruitment year (quintiles), time between recruitment and the first pregnancy or GDM (continuous), number of pregnancies during follow-up (discrete), parity (discrete), multiple pregnancies (discrete), time spent watching TV (tertiles), hypertension (discrete), following a nutritional therapy (discrete), and energy intake (tertiles).

**Table 4:** Sensitivity analysis.

		Tertiles of Consumption			
	No. Cases/Total	T1	T2	T3	P for Trend
**Pooled sample**					
Overall	186/3730	Reference	1.41 [0.96, 2.06]	1.10 [0.74, 1.64]	0.818
Excluding prevalent cases of CVD and cancer	183/3671	Reference	1.35 [0.92, 1.98]	1.11 [0.74, 1.65]	0.749
Changing the energy limits (≥1000 kcal and ≤3500 kcal)	160/3338	Reference	1.42 [0.94, 2.14]	1.19 [0.77, 1.84]	0.560
Excluding women following a nutritional therapy	170/3482	Reference	1.30 [0.88, 1.93]	1.10 [0.72, 1.65]	0.796
Excluding women with past pregnancies	161/3015	Reference	1.47 [0.97, 2.24]	1.16 [0.75, 1.79]	0.670
Excluding women whose first pregnancy was 10 years after recruitment	147/3232	Reference	1.20 [0.78, 1.84]	1.15 [0.76, 1.75]	0.506
Adjusting for adherence to the Mediterranean diet	186/3730	Reference	1.41 [0.96, 2.08]	1.10 [0.74, 1.65]	0.824
Adjusting for carbohydrate and saturated fat intake	186/3730	Reference	1.40 [0.95, 2.06]	1.09 [0.72, 1.64]	0.853
Using UPF baseline consumption if GDM or first pregnancy was before 10 years	186/3730	Reference	1.17 [0.80, 1.72]	1.04 [0.71, 1.53]	0.908
Using %UPF of energy intake instead of servings/day	186/3730	Reference	1.09 [0.74, 1.61]	1.09 [0.73, 1.63]	0.682
**Women <30 years**					
Overall	124/2538	Reference	1.25 [0.79, 1.98]	0.89 [0.54, 1.46]	0.524
Excluding prevalent cases of CVD and cancer	122/2505	Reference	1.20 [0.75, 1.91]	0.89 [0.54, 1.46]	0.543
Changing theenergy limits (≥1000 kcal and ≤3500 kcal)	107/2264	Reference	1.12 [0.69, 1.84]	0.95 [0.55, 1.63]	0.800
Excluding women following a nutritional therapy	113/2383	Reference	1.16 [0.73, 1.86]	0.84 [0.50, 1.42]	0.451
Excluding women with past pregnancies	120/2341	Reference	1.26 [0.79, 2.02]	0.92 [0.55, 1.55]	0.647
Excluding women whose first pregnancy was 10 years after recruitment	90/2098	Reference	1.06 [0.63, 1.79]	0.83 [0.47, 1.47]	0.499
Adjusting for adherence to the Mediterranean diet	124/2538	Reference	1.24 [0.78, 1.98]	0.89 [0.53, 1.47]	0.522
Adjusting for carbohydrate and saturated fat intake	124/2538	Reference	1.24 [0.78, 1.97]	0.87 [0.52, 1.45]	0.480
Using UPF baseline consumption if GDM or first pregnancy was before 10 years	124/2538	Reference	1.14 [0.72, 1.80]	0.88 [0.54, 1.43]	0.545
Using %UPF of energy intake instead of servings/day	124/2538	Reference	0.95 [0.60, 1.49]	0.83 [0.51, 1.35]	0.439
**Women ≥30 years**					
Overall	62/1192	Reference	1.56 [0.77, 3.15]	2.05 [1.03, 4.07]	0.041
Excluding prevalent cases of CVD and cancer	61/1166	Reference	1.50 [0.73, 3.06]	2.05 [1.02, 4.10]	0.042
Changing the energy limits (≥1000 kcal and ≤3500 kcal)	53/1074	Reference	2.04 [0.91, 4.60]	2.36 [1.04, 5.36]	0.045
Excluding women following a nutritional therapy	57/1099	Reference	1.49 [0.71, 3.13]	2.21 [1.08, 4.55]	0.028
Excluding women with past pregnancies	41/674	Reference	1.85 [0.71, 4.83]	3.23 [1.27, 8.22]	0.011
Excluding women whose first pregnancy was 10 years after recruitment	53/1134	Reference	1.48 [0.69, 3.21]	2.50 [1.20, 5.22]	0.011
Adjusting for adherence to the Mediterranean diet	62/1192	Reference	1.57 [0.78, 3.14]	2.06 [1.05, 4.06]	0.039
Adjusting for carbohydrate and saturated fat intake	62/1192	Reference	1.61 [0.78, 3.30]	2.16 [1.06, 4.42]	0.034
Using UPF baseline consumption if GDM or first pregnancy was before 10 years	62/1192	Reference	1.26 [0.62, 2.59]	1.55 [0.81, 2.97]	0.180
Using %UPF of energy intake instead of servings/day	62/1192	Reference	0.92 [0.44, 1.91]	1.52 [0.77, 3.01]	0.208

Values are odd ratios (OR) and 95% confidence intervals. The model was adjusted for age (continuous), BMI (continuous), education (discrete), smoking status (discrete), physical activity (tertiles), family history of diabetes (discrete), recruitment year (quintiles), time between recruitment and first pregnancy or GDM (continuous), number of pregnancies during follow-up (discrete), parity (discrete), multiple pregnancies (discrete), time spent watching TV (tertiles), hypertension (discrete), following a nutritional therapy (discrete), and energy intake (tertiles).

## Data Availability

The data presented in this study are available on request from the corresponding author (alessandro.leone1@unimi.it) and Maira Bes-Rastrollo (mbes@unav.es).
